# Community-based insights into the connection between endocrine-disrupting chemicals and depressive symptoms

**DOI:** 10.1016/j.crtox.2025.100225

**Published:** 2025-02-25

**Authors:** Yun-An Liu, Heng-Jung Hsu, Heng-Chih Pan, Chiao-Yin Sun, Yih-Ting Chen, Chin-Chan Lee, Feng-Chieh Su, Yi-Chia Wei, Cheng-Kai Hsu, Chun-Yu Chen

**Affiliations:** a Department of Nephrology, Chang Gung Memorial Hospital, Keelung Branch 222, Mai-Chin Road, Keelung 20401, Taiwan, Republic of China; b College of Medicine, Chang Gung University, Taoyuan, Taiwan; c Community Medicine Research Center, Chang Gung Memorial Hospital, Keelung Branch 222, Mai-Chin Road, Keelung 20401, Taiwan, Republic of China; d Department of Neurology, Chang Gung Memorial Hospital, Keelung Branch 222, Mai-Chin Road, Keelung 20401, No. 259, Wenhua 1st Rd., Guishan Dist., Taoyuan City 33302, Taiwan, Republic of China; e School of Traditional Chinese Medicine, College of Medicine, Chang Gung University, No. 259, Wenhua 1st Rd., Guishan Dist., Taoyuan City 33302, Taiwan, Republic of China

**Keywords:** Endocrine-disrupting chemicals, Depressive symptoms, Paraben, Phthalate, Community medicine, Environmental hormone

## Abstract

**Background:**

The rising prevalence of depressive disorders has sparked concerns regarding environmental risk factors, particularly exposure to endocrine-disrupting chemicals (EDCs). However, the link between EDC exposure and depressive symptoms remains largely unexplored.

**Methods:**

The Chang Gung Community Medicine Research Center carried out a cross-sectional study across four regions in northeastern Taiwan. Out of 887 participants, 120 subjects were chosen according to their EDC exposure scores. These participants underwent urinary EDC analysis and were evaluated for depressive symptoms through the standardized Hospital Anxiety and Depression Scale − Depression subscale (HADS-D) questionnaire.

**Results:**

Participants with HADS-D scores ≥ 8 exhibited significantly higher EDC exposure score compared to those with lower scores. The correlation analyses identified a notible positive association between urinary monobenzyl phthalate (MBzP) levels and HADS-D scores (*r* = 0.244, *p* = 0.007). Multiple regression analysis revealed that MBzP was independently linked to increased HADS-D scores in a positive manner (β ± SE: 0.139 ± 0.050, *p* = 0.006). Multivariable logistic regression indicated that higher MBzP (OR: 1.150, 95 % CI: 1.036–1.278, *p* = 0.009) and methylparaben (MP) levels (OR: 1.008, 95 % CI: 1.003–1.013, p < 0.001) showed a significant correlation with the likelihood of HADS-D scores ≥ 8. Receiver operating characteristic curve analysis demonstrated that elevated levels of MBzP, MP and the EDCs exposure score were associated with a greater likelihood of depressive symptoms.

**Conclusion:**

Exposure to EDCs, particularly MBzP and MP, could be associated with a heightened risk of depressive symptoms.

## Highlights

• EDC exposure is significantly linked to depressive symptoms, highlighting its role as a mental health risk factor.
• Study reveals potential mental health risks from common environmental toxins.
• Elevated Monobenzyl phthalate (MBzP), Methylparaben (MP), and EDC exposure scores are associated with depressive symptoms.
• Findings emphasize the need for public awareness of environmental hormone exposure effects.

## Graphical abstract

**Figure:**
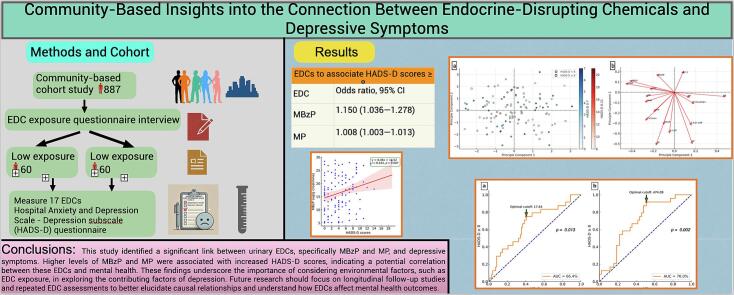

## 1 Introduction

Endocrine-disrupting chemicals (EDCs) are artificial substances that disrupt the human endocrine system by imitating the properties of natural chemical messengers, especially estrogen (Hu et al., 2009). EDCs are frequently present in industrial, household, agricultural, and healthcare environments (Kabir et al., 2015, Park et al., 2022).

The growing production and environmental discharge of these substances present a significant risk to ecological stability and human well-being. These substances comprise both artificial and naturally derived chemicals, including plasticizers, phytoestrogens, organochlorine pesticides, dioxins, and medicinal compounds. A significant number of EDCs have been associated with a range of health problems, including obesity, kidney disease, asthma, premature thelarche, cryptorchidism, and neurocognitive disorders (Bornehag et al., 2004, Cai et al., 2019, Colon et al., 2000, Duty et al., 2003). These issues underscore the need for public health initiatives focused on minimizing EDC exposure (Yilmaz et al., 2020).

Phthalates, a specific category of EDCs, are extensively utilized as plasticizers in industrial settings to enhance the elasticity, adaptability, and resilience of polyvinyl chloride (PVC). Di-(2-ethylhexyl) phthalate (DEHP), a long-chain phthalate commonly found in PVC materials, can gradually leach into the environment, making it a frequent environmental contaminant (Dutta et al., 2020, Lu et al., 2014). Human are exposed to DEHP through skin contact with various items, as well as through inhalation or ingestion, including food containers, cosmetic products, and toys, or through bloodstream exposure from medical devices, including hemodialysis circuits, blood transfusions, cardiopulmonary bypass systems, and other intensive care applications involving extensive tubing. DEHP has been linked to kidney damage through both animal studies and epidemiological research, contributing to conditions such as interstitial fibrosis, albuminuria, podocyte injury, glomerulosclerosis, and disruption of the intrarenal renin-angiotensin system (Tsai et al., 2016, Wei et al., 2012).

Gonadal hormones play a critical role in neurodevelopment and cognitive function by modulating essential neurotransmitter systems, supporting neuron survival, contributing to myelination, and influencing cognitive processes in the aging brain, particularly in relation to learning and memory (Janowsky, 2006, Schumacher et al., 2003, Weiss, 2007). Disruption of these hormonal pathways can have profound neurological consequences. EDCs interfere with these processes by mimicking or antagonizing the actions of androgens, estrogens, anti-estrogens, and anti-androgens, thereby impeding neurogenesis and altering neural network function. This hormonal interference renders the brain especially vulnerable to EDC exposure, particularly during gestation, potentially leading to alterations in neurotransmitter regulation and affecting offspring's learning, memory, and social behaviors (Salazar et al., 2021, Weiss, 2007).

In addition, studies have shown that EDCs can also affect neurobehavioral outcomes, including neurocognitive dysfunction, further highlighting their impact on cognitive and emotional development (Chang et al., 2021, de Cock et al., 2012, Park et al., 2015, Salazar et al., 2021). Depression is also one of the associated outcomes, as evidenced by findings from animal studies (Xu et al., 2015, Zuo et al., 2014). In humans, certain urinary phthalate metabolites, including mono-(n-butyl) phthalate (MnBP), Mono-(3-carboxypropyl) phthalate (MCPP), and Mono-(carboxynonyl) phthalate (MCNP), have been identified as being related to a heightened risk of depression (Kim et al., 2016). Additionally, another study identified a similar association with DEHP metabolites, such as, mono-(2-ethyl-5-hydroxyhexyl) phthalate (MEHHP), mono-(2-ethyl-5-oxohexyl) phthalate (MEOHP), and mono-(2-ethylhexyl) phthalate (MEHP), further supporting the potential link between phthalate exposure and depressive symptoms (Kim et al., 2016, Lee et al., 2018). EDCs have been linked to increased oxidative stress indices in brain tissue, and the association between oxidative stress in the brain and depression has been highlighted in numerous studies (Ait Tayeb et al., 2023, Bajpai et al., 2014, Ferguson et al., 2015, Hong et al., 2009, Zuo et al., 2014). This connection is possibly related to dysregulation in the serotonin pathways, neurogenesis, stress response and neuroinflammation, as well as imbalances in synaptic plasticity (Bonaccorso et al., 2002, Correia et al., 2023, Troubat et al., 2021).

Depressive disorder is a widespread and debilitating condition in contemporary industrialized societies, currently ranked as the 13th primary global cause of disability and death, with an occurrence rate of 12 % over a person's lifetime (Diseases and Injuries, 2020). Major depressive disorder (MDD) is considered to have a multifactorial etiology, encompassing neurobiological, inflammatory, genetic, environmental, and psychosocial influences (Cui et al., 2024). Traditionally, MDD was primarily thought to arise from neurotransmitter imbalances, particularly involving serotonin, norepinephrine, and dopamine (Bhatt et al., 2021). However, current understanding acknowledges the complexity of its origins and the significance of additional factors. One such factor is the impact of EDCs. EDCs not only affect neurotransmitters like dopamine and serotonin but also contribute to oxidative stress and inflammatory responses—processes that are all implicated in the development of mood disorders, including depression (Hamid et al., 2021). Furthermore, EDCs can interfere with both the hypothalamic–pituitary–adrenal (HPA) axis, disrupting its feedback system, and affect gonadal hormones, both of which are crucial in regulating the body's response to stress (Egalini et al., 2022, Keller et al., 2017, Pariante and Lightman, 2008, Potzl et al., 2024, Quinnies et al., 2015, Vreeburg et al., 2009). Insulin resistance has been linked to depression, with potential contributions from inflammatory responses and the dysregulation of the HPA axis (Bai et al., 2018, Fernandes et al., 2022, Watson et al., 2018). This connection is further supported by findings on exposure to EDCs, which is associated with an increased risk of diabetes mellitus, as they are known to induce insulin resistance, promote chronic inflammation, impair pancreatic beta-cell function, and disrupt immune system regulation (Howard, 2018, Lin and Yin, 2023, Rotondo and Chiarelli, 2020).

Certain types of EDCs, including BPA, Polybrominated diphenyl ethers (PBDEs), Polychlorinated biphenyls (PCBs), and phthalates, may disrupt endocrine cell function by altering or dysregulating hormone secretion through their interference with cellular signaling pathways (Egalin et al., 2022). Notably, DEHP has been specifically identified as affecting the HPA axis in one study (Quinnie et al., 2015). EDCs have also been shown to disrupt the homeostatic and stimulus-driven responses of thyroid hormone regulation, with involvement of the hypothalamic-pituitary-thyroid (HPT) axis in the underlying pathophysiology of depression, particularly due to its feedback relationship with serotoninergic systems and the presence of hypothyroidism within the brain (Egalini et al., 2022, Foltyn et al., 2002, Ibhazehiebo and Koibuchi, 2011, Jackson, 1998, Rotondo and Chiarelli, 2020, Schmutzler et al., 2007, Thambirajah et al., 2022). Disruptions in these systems have been linked to mood disorders, reinforcing the notion that EDC exposure may be a significant environmental factor in MDD. Recent research conducted in Korea and the United States has demonstrated that certain phthalates −common EDCs-are positively associated with a higher likelihood of depressive symptoms in the general population, particularly in adults later in life (Kim et al., 2016, Lee et al., 2018, Wang et al., 2023).

Significantly higher levels of phthalate plasticizer exposure than those in other countries have been reported in the Taiwanese population by several researches (Su et al., 2024, Zigmond and Snaith, 1983). Our previous community cohort studies in northern Taiwan revealed that phthalate exposure, particularly DEHP, is linked to a reduction in estimated glomerular filtration rate (eGFR), albuminuria, and neurocognitive decline. However, the relationship between phthalate exposure and depressive disorders among community residents has been the subject of limited research (Chen et al., 2024, Chen et al., 2021, Su et al., 2024). To address this gap, we launched a community-based cohort study across four northeastern districts of Taiwan. This study include annual community visits to involve participants in health evaluations, blood and urine tests, detailed surveys about EDC exposure and depressive symptoms, and urinary EDC measurements. The main objective of the research is to investigate the possible link between EDC exposure and depressive disorders.

## 2 Materials and methods

### 2.1 Study design

Since November 2013, the Community Medicine Research Center (CMRC) has been conducting a prospective cohort study in the Gongliao, Ruifang, and Wanli districts, along with Keelung City in northern Taiwan. This ongoing study seeks to detect potential health issues at an early stage and promptly address them through regular health evaluations, providing significant benefits to the community. The study encompasses various activities, including blood sampling, urine analyses, physical check-ups, and surveys on health-related behaviors, along with educational seminars on health, hygiene instruction, community outreach programs, and engaging activities.

### 2.2 Participants and public engagement

Health stations or community centers in different districts served as venues for the annual outreach activities. Community residents were selected as participants and were expected to engage in the activities at intervals of 2–3 years. In 2019, an EDC survey was incorporated into the program. Participants eligible for inclusion were individuals aged 18 and older who had participated in the health activities and agreed to undergo an EDC assessment and complete a questionnaire interview. Each participant gave informed consent before being included in the study.

**Fig. 1:**
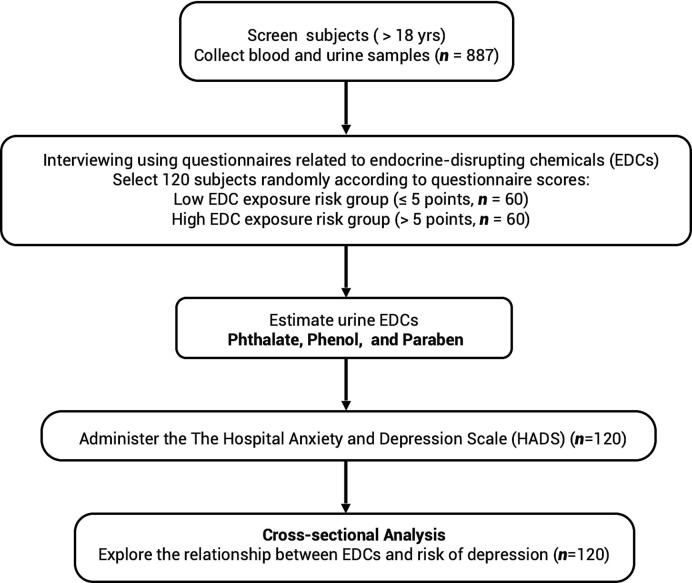
Flowchart depicting participant selection for urinary endocrine-disrupting chemical assessment.

A total of 887 participants completed the EDC-focused questionnaire and laboratory testing. From this cohort, 120 individuals were selected for further study. This selection included 60 participants identified as having low EDC exposure risk (questionnaire scores ≤ 5 points) and 60 categorized as high risk (questionnaire scores > 5 points). These participants underwent an in-depth evaluation of urinary EDC concentrations and were assessed for depressive symptoms through the Hospital Anxiety and Depression Scale − Depression subscale (HADS-D) questionnaire (Fig. 1). A team of trained interviewers administered a standardized questionnaire to all participants, collecting detailed information on personal medical history, familial disease history, participants' occupation, exercise habits, tobacco use, educational background, living conditions, alcohol consumption, and lifestyle choices that could increase the likelihood of EDC exposure, cognitive decline, anxiety and depressive symptoms.

Participant recruitment and sample preservation were fundamental components of the Northeastern Taiwan Community Medicine Research Cohort (NTCMRC, ClinicalTrials.gov Identifier: NCT04839796). The study strictly followed the principles of the Declaration of Helsinki and received approval from the Ethics Committee of the Institutional Review Board at Chang Gung Memorial Hospital (IRB: 201800275B0C602).

### 2.3 Questionnaires concerning EDC exposure and potential cognitive impairment

Seven questionnaires were developed by the CMRC to collect detailed information on participants' exposure to EDCs (Table S1). Moreover, the Hospital Anxiety and Depression Scale − Depression subscale (HADS-D), introduced in 1983 by Zigmond and Snaith, was designed with seven items to detect and assess depressive disorders (Table S2) (Zigmond and Snaith, 1983). The HADS-D screening tool includes seven questions, with score ranges indicating normal emotional status (0–7), borderline abnormality (8–10), and abnormal status (11–21). Ten interviewers were trained to administer the questionnaires, gather data on EDC-contaminated food consumption, evaluate lifestyle practices, and prioritize the HADS-D questionnaire. Respondents were additionally asked to report any observed changes in the participant.

### 2.4 Collection and processing of serum and urine samples

Participants were required to fast overnight before providing blood samples, which were promptly transported to the laboratory for analysis within four hours of collection. Biochemical tests and complete blood counts were performed on these samples. Following this, a portion of the samples was centrifuged at 3000×*g* for 10 min at 4 °C in refrigerated tubes to separate the serum. The serum was then subjected to further testing, with samples showing signs of lipemia or hemolysis excluded from the study. The remaining serum was aliquoted and stored at −80 °C for future analysis. Additionally, morning urine samples were collected from participants using the midstream clean-catch method.

### 2.5 Analysis of EDCs

The urinary samples underwent comprehensive analysis to detect a variety of EDCs, including phenols, parabens, and phthalates. The analysis focused on the following EDCs and their metabolites: (1) Phthalate metabolites, such as monomethyl phthalate (MMP), monoethyl phthalate (MEP), mono-(n-butyl) phthalate (MnBP), monobenzyl phthalate (MBzP), mono-(2-ethylhexyl) phthalate (MEHP), and mono-*iso*-nonyl phthalate (MiNP); (2) Phenols, including bisphenol A (BPA), nonylphenol (NP), 4-*tert*-octylphenol (4-t-OP), and 2,4-di-*tert*-butylphenol (2,4-di-t-BP), as well as triclosan and triclocarban; (3) Parabens, such as methylparaben (MP), ethylparaben (EP), propylparaben (PP), and butylparaben (BP); (4) Other compounds, including benzophenone-3 (BP-3).

Urine samples were thawed at 4 °C over a 24-hour period. For compound extraction, 100 μL of urine was combined with 20 μL of methanol (MeOH) containing five stable isotope-labeled internal standards (D_7_-PP, ^13^C_4_-MEHP, ^13^C_4_-MiNP, ^13^C_12_-BPA and ^13^C_6_-4-t-OP), along with 5 μL of β-glucuronidase (85,000 U/mL) and 20 μL of 1 M ammonium acetate solution. The mixture was prepared in a 1.5-mL microcentrifuge tube. The mixture was thoroughly vortexed for 10 s using a Vortex-2 Genie shaker (Scientific Industries, USA) to ensure homogeneity, followed by a one-hour incubation at 40 °C. To enhance extraction, 135 μL of 0.1 % formic acid in water was added, and the solution was processed through a supported liquid extraction cartridge (Isolute® SLE + 400 μL Capacity SLE Column, Biotage). Elution was performed using 2.0 mL of dichloromethane, after which the extract was evaporated to dryness and reconstituted in 200 μL of a 1:1 methanol–water solution. The final sample was analyzed employing UPLC-MS/MS.

The analysis of 17 specific EDCs in human urine samples was conducted using a Waters ACQUITY UPLC system coupled with a SCIEX API-4000 triple quadrupole mass spectrometer with an electrospray ionization source. Compounds were identified and quantified in multiple reaction monitoring (MRM) mode. BP-3 was analyzed in positive ionization, while the other 16 analytes were in negative ionization mode. Chromatographic separation used a Thermo Scientific Syncronis C^18^ column (150 × 2.1 mm, 3 μm). The mobile phases included 0.1 % formic acid in Milli-Q water (MPA-1), Milli-Q water (MPA-2), and acetonitrile (MPB-1). Twelve analytes—including phthalates, parabens, triclosan, and triclocarban—were separated using MPA-1 and MPB-1, while BPA, BP-3, and alkyl phenols used MPA-2 and MPB-1. The column temperature was maintained at 30 °C, the sample tray at 4 °C, and the injection volume was 10 μL. Data were acquired using Analyst 1.6.2 software (SCIEX Corporation).

### 2.6 Validation of EDC measurement method

This study evaluated the analytical performance of the UPLC-MS/MS method for detecting 17 EDC analytes in urine samples, focusing on linearity, limits of quantification (LOQs), and quality control metrics. A matrix-matched calibration using six levels with synthetic urine was applied. Linearity was assessed using the coefficient of determination (R^2^), and stable isotope-labeled internal standards were used to adjust retention times. The linear range varied: 10–1500 ng/mL for MEP, MP, triclosan, triclocarban, and benzophenone-3; 0.10–50.00 ng/mL for 4-t-OP and NP; and 1–500 ng/mL for the remaining analytes. Calibration curves for all analytes followed a weighted (1/x) linear model with R^2^ values > 0.990. LOQs were 0.10 ng/mL for NP and 4-t-OP, and 0.3 ng/mL for others. Precision and accuracy were assessed intra-day and inter-day, meeting European Medicines Agency and EU guidelines (European Medicines Agency, 2011).

### 2.7 Outcomes

This study sought to examine the link between urinary EDC concentrations and the risk of depressive disorder in participants, using the HADS-D questionnaire for depression screening. The analysis included clinical characteristics, estimated glomerular filtration rate, vitamin D levels, demographic details, and EDC exposure data in conjunction with the HADS-D scores.

### 2.8 Statistical analysis

The normality of continuous variables was determined using the Kolmogorov-Smirnov test, as well as skewness and kurtosis measures. Demographic and clinical characteristics between groups with HADS-D scores ≥ 8 and < 8 were compared via Student's *t*-test for normally distributed variables, and the Mann-Whitney *U* test for those without normal distribution. Categorical variables were assessed using the chi-square test.

To assess the correlation between HADS-D scores and EDC concentrations, one-way analysis of variance (ANOVA) was conducted to analyze trends in EDC levels across the three HADS-D severity categories. Multiple linear regression, adjusting for age, gender, and diabetes, was used to evaluate the linear association. Odds ratios for predicting HADS-D scores ≥ 8 were estimated using binary logistic regression, with Model 1 adjusted for age and gender, and Model 2 adjusted for all relevant variables.

A correlation matrix and Principal Component Analysis (PCA) were used to assess the relationships between EDCs and HADS-D scores. The correlation matrix identified linear associations between individual EDCs and depressive symptoms, revealing positive or negative correlations. PCA was applied to reduce data dimensionality and address multicollinearity, transforming the EDCs into principal components that capture the most variance. This allowed for the identification of broader patterns and relationships between chemical exposure profiles and depressive symptoms.

Receiver operating characteristic (ROC) curves were utilized to examine the relationship between MBzP, MP, body mass index, or EDC exposure scores and occurrences of HADS-D ≥ 8. The discriminative ability of these variables was assessed using the area under the ROC curve (AUC). ROC curves, applying Youden's index, were used to establish the cut-off values for each variable.

All statistical analyses were two-tailed, with significance set at a p-value of < 0.05. Data analysis was carried out using SPSS software version 27.0 for Mac, while graphs were generated with GraphPad Prism version 10, SPSS, and Stata/MP 15.1 for Mac.

## 3 Results

### 3.1 Participant demographics

Within the 120 participants, those with HADS-D scores ≥ 8 exhibited comparable age, gender distribution, albumin levels, and serum vitamin D concentrations compared to the group with HADS-D scores < 8 [49.83 ± 15.43 vs. 54.80 ± 13.49 years, *p* = 0.641; 41.7 % vs. 36.5 % male, *p* = 0.637; 4.66 ± 0.26 vs. 4.70 ± 0.24 g/dL, *p* = 0.515; 28.63 ± 10.82 vs. 26.93 ± 9.21 ng/mL] (Table 1). The prevalence of diabetes, dyslipidemia, and chronic kidney disease was likewise similar between the two groups [16.7 % vs. 16.7 %, *p* = 1.000; 70.8 % vs. 85.4 %, *p* = 0.093; 33.3 % vs. 31.3 %, *p* = 0.844]. However, the group with HADS-D scores ≥ 8 demonstrated appreciably higher levels of homeostasis model assessment of insulin resistance index, insulin, and EDC exposure scores [16.86 ± 10.69 vs. 10.93 ± 7.66 μU/mL, *p* = 0.002; 4.97 ± 4.55 vs. 2.88 ± 2.58, *p* = 0.003; 6.67 ± 4.72 vs. 4.38 ± 4.26 points, *p* = 0.023].

**Table 1:** Demographics and clinical characteristics of study cohorts testing the urinary EDCs.

	HADS-D score ≥ 8 (n = 24)	HADS-D score < 8 (n = 96)	*p* value
Age (years)	49.83 ± 15.43	54.80 ± 13.49	0.641
Male, n, (%)	10 (41.7)	35 (36.5)	0.637
HADS-D score	10.04 ± 2.49	3.03 ± 2.33	< 0.001*
EDC exp score	6.67 ± 4.72	4.38 ± 4.26	0.023*
EDC exp > 5, n, (%)	15 (62.5)	45 (46.9)	0.171
Diabetes, n, (%)	4 (16.7)	16 (16.7)	1.000
Dyslipidemia, n, (%)	17 (70.8)	82 (85.4)	0.093
CKD,n, (%)	8 (33.3)	30 (31.3)	0.844
Hypertension, n, (%)	13 (54.2)	57 (59.4)	0.643
White blood cell (1000/μL)	6.10 ± 1.69	6.02 ± 1.68	0.850
Hemoglobin(g/dL)	13.89 ± 1.62	14.09 ± 1.54	0.575
Ferritin (ng/mL)	276.73 ± 290.29	198.44 ± 9 ± 159.05	0.076
Folate(ng/mL)	10.81 ± 6.37	11.54 ± 7.63	0.669
AST (U/L)	23.46 ± 9.28	21.10 ± 6.05	0.132
Total bilirubin (mg/dL)	0.58 ± 0.23	0.63 ± 0.29	0.438
Insulin (uU/mL)	16.86 ± 10.69	10.93 ± 7.66	0.002*
LDL (mg/dL)	124.83 ± 32.02	128.68 ± 34.03	0.623
HS-CRP (mg/L)	3.36 ± 6.58	1.87 ± 2.95	0.122
Bun (mg/dL)	13.49 ± 3.27	14.54 ± 5.12	0.341
Creatinine (mg/dL)	0.77 ± 0.14	0.80 ± 0.24	0.490
eGFR (mL/min/1.73 m^2^)	95.38 ± 21.89	89.53 ± 19.40	0.200
Na (mEq/L)	141.13 ± 2.35	141.54 ± 1.62	0.308
K (mEq/L)	4.29 ± 0.34	4.44 ± 0.35	0.061
Ca (mg/dL)	9.49 ± 0.33	9.48 ± 0.33	0.925
P (mg/dL)	3.65 ± 0.52	3.66 ± 0.50	0.936
Albumin (g/dL)	4.66 ± 0.26	4.70 ± 0.24	0.515
Vitamin D (ng/mL)	28.63 ± 10.82	26.93 ± 9.21	0.438
Vitamin B12 (pg/mL)	702.59 ± 724.40	674.07 ± 438.70	0.806
Folate(ng/mL)	10.81 ± 6.37	11.54 ± 7.63	0.669
Body-mass index	26.76 ± 4.48	24.25 ± 4.61	0.150
UACR (mg/g)	5.60 (3.78–21.58)	8.10 (3.65–20.68)	0.691
UPCR (mg/g)	65.83 (48.44–95.79)	60.61 (51.06–84.25)	0.809
HOMA-IR	4.97 ± 4.55	2.88 ± 2.58	0.003*
Leptin (ng/mL)	12.02 ± 7.68	11.84 ± 9.00	0.957

Abbreviations: EDCs, endocrine-disrupting chemicals; HADS-D, Hospital Anxiety and Depression Scale − depression subscale; EDC exp, EDC exposure questionnaire; CKD, chronic kidney disease; AST, aspartate transaminase; ALT, alanine transaminase; HS-CRP, high sensitivity C-reactive protein; eGFR, estimated glomerular filtration rate; UACR, urine albumin-to-creatinine ratio; UPCR, urine protein-to-creatinine ratio; HOMA-IR, homeostasis model assessment-insulin resistance index.

*: *p* value < 0.05.

### 3.2 PCA of EDCs and HADS-D scores

**Fig. 2:**
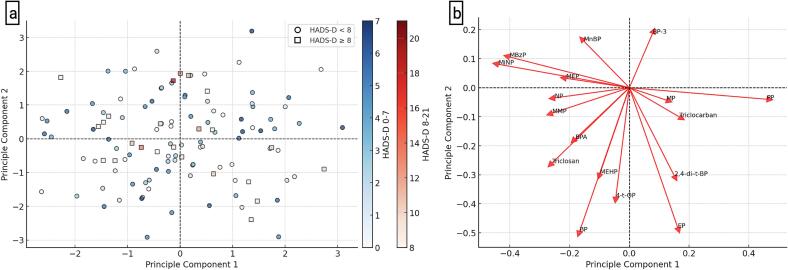
Panel a: Principal Component Analysis (PCA) Plot with HADS-D Classification. This Principal Component Analysis (PCA) plot illustrates the distribution of individuals based on the levels of 17 endocrine-disrupting chemicals (EDCs). Each data point represents an individual, with color gradients indicating the HADS-D score. Two distinct markers differentiate the groups: Open circles (○, blue-shaded) represent individuals with HADS-D < 8. Open squares (□, red-shaded) represent individuals with HADS-D ≥ 8. The two color bars on the right indicate the gradient of HADS-D scores: The blue color bar (0–7 points) corresponds to lower HADS-D scores. The red color bar (8–21 points) corresponds to higher HADS-D scores. Dashed black lines at PC1 = 0 and PC2 = 0 divide the plot into four quadrants, aiding in data interpretation. This visualization aims to identify potential clustering patterns of individuals based on EDC exposure and their association with depressive symptoms Panel b: PCA Loadings Plot for EDCs. This PCA loadings plot illustrates the contribution of 17 EDCs to the PC1 and PC2. Each red arrow represents a specific chemical, indicating its loading direction and magnitude. The length of the arrow reflects the degree of influence that the corresponding EDC has on the principal components. Longer arrows indicate greater contributions of the chemical to the PCA dimensions. Arrows pointing in similar directions suggest positive correlations between chemicals. Opposing arrows indicate negative correlations between variables. This figure provides insight into how different EDCs drive the variance within the PCA space and aims in understanding their associations with HADS-D scores and potential mental health risks. (For interpretation of the references to color in this figure legend, the reader is referred to the web version of this article.)

In this study, we conducted a PCA on 17 EDCs and HADS-D scores to explore the potential association between EDC exposure and depressive symptoms. The PCA score plot (Fig. 2a) presents the distribution of individual participants along the first two principal components (PC1 and PC2), which together capture the primary variance within the dataset. The PCA score plot indicates a potential clustering pattern, with participants having higher HADS-D scores (≥8) preferentially occupying specific regions of the PCA space, suggesting a possible link between distinct EDC exposure profiles and depressive symptoms. In contrast, individuals with HADS-D < 8 exhibit a more dispersed distribution, implying greater variability in their exposure patterns. The PCA loadings plot (Fig. 2b) illustrates the contribution of each EDC variable to the first two principal components. Each red arrow represents a specific chemical, with its direction and length reflecting the magnitude and orientation of its influence in the PCA space. Several EDCs exhibit strong contributions to PC1 and PC2, suggesting that specific exposures may drive the observed separation in the PCA score plot. The alignment of certain chemicals with the clustering pattern of individuals with higher HADS-D scores further supports a potential association between EDC exposure and depressive symptoms.

### 3.3 Correlation and regression analysis of EDCs with depressive symptoms

**Fig. 3:**
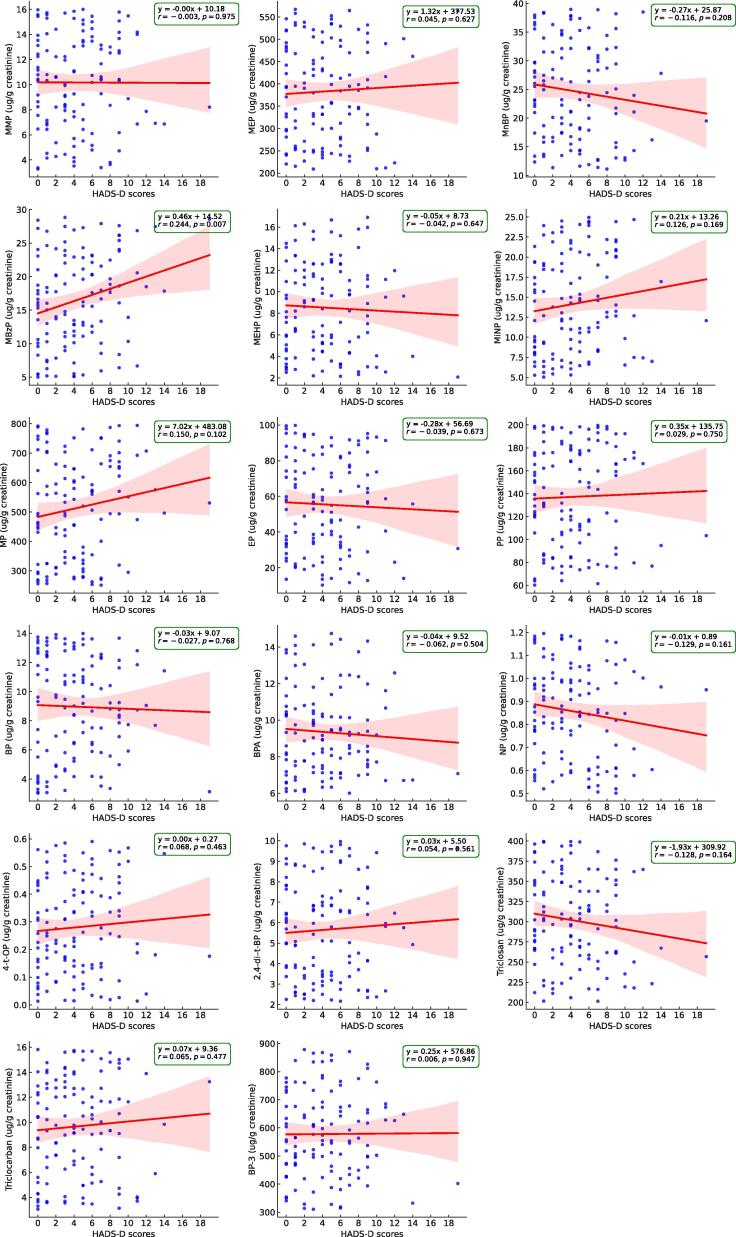
Scatter plots illustrating the associations between urinary endocrine-disrupting chemical (EDC) concentrations and Hospital Anxiety and Depression Scale–Depression subscale (HADS-D) scores. The red line represents the regression line of the data points (blue dots), and the red shaded area represents the 95% confidence interval of the regression line. Abbreviations: MMP, monomethyl phthalate; MEP, monoethyl phthalate; MnBP, mono-n-butyl phthalate; MBzP, monobenzyl phthalate; MEHP, mono-(2-ethylhexyl) phthalate; MiNP, monoisononyl phthalate; BPA, bisphenol A; NP, nonylphenol; 4-t-OP, 4-*tert*-octylphenol; 2,4-di-t-BP, 2,4-di-*tert*-butylphenol; MP, methylparaben; EP, ethylparaben; PP, propylparaben; BP, butylparaben; BP-3, benzophenone-3. (For interpretation of the references to color in this figure legend, the reader is referred to the web version of this article.)

Besides, individuals were categorized into three groups according to the HADS-D score definitions of depression severity and analyzed using one-way ANOVA (Table 2). Although the 17 EDCs did not exhibit a significant stepwise trend, MBzP and MP displayed varying concentrations that appeared to increase with higher HADS-D scores, despite not reaching statistical significance (*p* for trend = 0.054 and 0.068, respectively). Fig. 3 presents the Pearson correlation coefficients (*r*), depicting the associations between the 17 EDCs and HADS-D scores. MBzP was found to be significantly correlated with an increase in HADS-D scores (*r* = 0.244, *p* = 0.007). However, the relationships of MEHP, MP, NP, 4-t-OP, and BP-3 with HADS-D scores were quantified as −0.042 (*p* = 0.647), 0.150 (*p* = 0.102), −0.129 (*p* = 0.161), 0.068 (*p* = 0.463), and 0.006 (*p* = 0.947), respectively. Our previous cross-sectional and longitudinal studies have established links between these five compounds and both kidney function deterioration and albuminuria, and MEHP was previously found to be associated with neurocognitive decline in an elderly population in another cross-sectional research(Chen et al., 2024, Chen et al., 2021). Simple linear regression analysis demonstrated that younger age and elevated MBzP levels were significantly correlated with increased HADS-D scores (β ± SE: −0.060 ± 0.024, *p* = 0.013; β ± SE: 0.131 ± 0.048, *p* = 0.007) (Table 3).

**Table 2:** Urinary EDCs of study cohorts.

	Overall (*n* = 120)	HADS-D ≤ 7 (*n* = 96, normal)	8 ≤ HADS-D ≤ 10 (*n* = 17, borderline abnormal)	HADS-D ≥ 11 (*n* = 7, abnormal)	*p* for trend
Urinary XEs (ug/g creatinine)					
MMP	9.370 (8.667–10.13)	9.354 (8.571–10.21)	9.695 (7.545–12.46)	8.827 (6.521–11.95)	0.552
MEP	367.5 (348.3–387.8	366.5 (345.2–389.1)	366.5 (317.5–422.9)	385.2 (265.6–558.5)	0.519
MnBP	23.16 (21.67–24.76)	23.48 (21.81–25.27)	21.96 (17.71–27.22)	21.94 (16.11–29.89)	0.566
MBzP	14.90 (13.63–16.28)	14.18 (12.83–15.67)	17.73 (14.28–22.02)	19.18 (12.03–30.59)	0.054
MEHP	7.350 (6.619–8.162)	7.508 (6.713–8.397)	7.122 (5.032–10.08)	5.930 (2.984–11.78)	0.423
MiNP	12.83 (11.80–13.95)	12.40 (11.28–13.64)	16.32 (13.58–19.60)	11.40 (7.333–17.72)	0.624
BPA	9.064 (8.671–9.475)	9.084 (8.640–9.550)	9.171 (8.153–10.31)	8.547 (6.538–11.17)	0.591
NP	0.831 (0.794–0.869)	0.840 (0.799–0.883)	0.788 (0.679–0.915)	0.8084 (0.6398–1.021)	0.684
4-t-OP	0.209 (0.176–0.247)	0.208 (0.174–0.249)	0.268 (0.160–0.450)	0.1169 (0.034–0.3996)	0.230
2,4-di–t-BP	5.126 (4.716–5.571)	5.152 (4.692–5.656)	4.820 (3.712–6.259)	5.554 (3.895–7.918)	0.831
Triclosan	296.3 (286.4–306.5)	298.3 (287.1–309.9)	295.0 (270.0–322.4)	272.1 (223.9–330.6)	0.232
Triclocarban	8.740 (8.011–9.536)	8.734 (7.939–9.609)	9.770 (7.604–12.55)	6.728 (3.914–11.56)	0.230
MP	483.5 (452.9–516.3)	459.5 (426.5–495.1)	589.9 (510.3–681.9)	599.9 (499.7–720.3)	0.068
EP	47.98 (43.17–53.32)	47.33 (42.11–53.20)	56.82 (41.74–77.35)	38.32 (21.38–68.69)	0.347
PP	129.7 (121.8–138.2)	128.7 (119.9–138.1)	141.7 (118.7–169.1)	117.0 (83.13–164.7)	0.476
BP	8.185 (7.541–8.885)	8.198 (7.451–9.021)	7.973 (6.661–9.544)	8.536 (5.479–13.30)	0.881
BP-3	557.8 (531.0–585.9)	554.9 (524.3–587.2)	576.5 (510.9–650.5)	552.5 (422.8–722.1)	0.929

Notes: Data are presented as geometric mean (95% confidence interval).

Abbreviations: HADS-D, Hospital Anxiety and Depression Scale − depression subscale; EDCs, endocrine-disrupting chemicals; MMP, Monomethyl phthalate; MEP, Monoethyl phthalate; MnBP, Mono-(n-butyl) phthalate; MBzP, Monobenzyl phthalate; MEHP, Mono-(2-ethylhexyl) phthalate; MiNP, Monoisononyl phthalate; BPA, bisphenol A; NP, Nonylphenol; 4-t-OP, 4-*tert*-Octylphenol; 2,4-di-t-BP, 2,4-di-*tert*-butylpheno; MP, Methylparaben; EP, Ethylparaben; PP, Propylparaben; BP, Butylparaben; BP-3, benzophenone-3.

& High exposure: total score > 6 points, Low exposure: total score <= 5,

*: *p* value < 0.05.

**Table 3:** β-coefficient between HADS-D scores and independent variables.

	Simple linear regression		Multiple regression analysis, model 1		Multiple regression analysis, model 2	
	β ± SE	*p*	β ± SE	*p*	β ± SE	*p*
Age	−0.060 ± 0.024	0.013*	—	—	−0.079 ± 0.027	0.004*
Gender	0.267 ± 0.694	0.702	—	—	−0.040 ± 0.687	0.954
Diabetes	−0.460 ± 0.901	0.611	−0.093 ± 0.900	0.918	−0.210 ± 0.945	0.825
MMP	−0.003 ± 0.090	0.975	−0.010 ± 0.089	0.906	−0.005 ± 0.093	0.957
MEP	0.002 ± 0.003	0.627	0.001 ± 0.003	0.779	−1.06*10^5^ ± 0.003	0.996
MnBP	−0.050 ± 0.040	0.208	−0.046 ± 0.039	0.241	−0.040 ± 0.040	0.322
MBzP	0.131 ± 0.048	0.007*	0.130 ± 0.047	0.007*	0.139 ± 0.050	0.006*
MEHP	−0.037 ± 0.080	0.647	−0.069 ± 0.080	0.391	−0.030 ± 0.081	0.712
MiNP	0.077 ± 0.055	0.169	0.053 ± 0.056	0.349	0.066 ± 0.058	0.257
BPA	−0.096 ± 0.143	0.504	−0.259 ± 0.150	0.087	−0.316 ± 0.159	0.050
NP	−2.331 ± 1.653	0.161	−2.629 ± 1.628	0.109	−2.825 ± 1.675	0.095
4-t-OP	1.464 ± 1.988	0.463	1.271 ± 1.966	0.519	2.669 ± 2.191	0.226
2,4-di–t-BP	0.082 ± 0.141	0.561	0.161 ± 0.141	0.257	0.111 ± 0.149	0.460
Triclosan	−0.008 ± 0.006	0.164	−0.007 ± 0.006	0.227	−0.010 ± 0.006	0.125
Triclocarban	0.062 ± 0.086	0.477	0.063 ± 0.085	0.458	0.043 ± 0.088	0.627
MP	0.003 ± 0.002	0.102	0.003 ± 0.002	0.086	0.003 ± 0.002	0.177
EP	−0.005 ± 0.013	0.673	−0.008 ± 0.013	0.536	−0.008 ± 0.014	0.543
PP	0.002 ± 0.008	0.750	0.003 ± 0.008	0.722	0.005 ± 0.008	0.535
BP	−0.029 ± 0.098	0.768	−0.035 ± 0.097	0.721	0.000 ± 0.104	0.543
BP-3	0.000 ± 0.002	0.947	0.000 ± 0.002	0.854	−0.002 ± 0.002	0.430

Abbreviations: HADS-D, Hospital Anxiety and Depression Scale − depression subscale; MMP, Monomethyl phthalate; MEP, Monoethyl phthalate; MnBP, Mono-(n-butyl) phthalate; MBzP, Monobenzyl phthalate; MEHP, Mono-(2-ethylhexyl) phthalate; MiNP, Monoisononyl phthalate; BPA, bisphenol A; NP, Nonylphenol; 4-t-OP, 4-*tert*-Octylphenol; 2,4-di-t-BP, 2,4-di-*tert*-butylpheno; MP, Methylparaben; EP, Ethylparaben; PP, Propylparaben; BP, Butylparaben; BP-3, benzophenone-3.

Model 1: adjusted for age and gender, Model 2: adjusted for age, gender, diabetes and 17 endocrine-disrupting chemicals.

*: *p* value < 0.05.

**Fig. 4:**
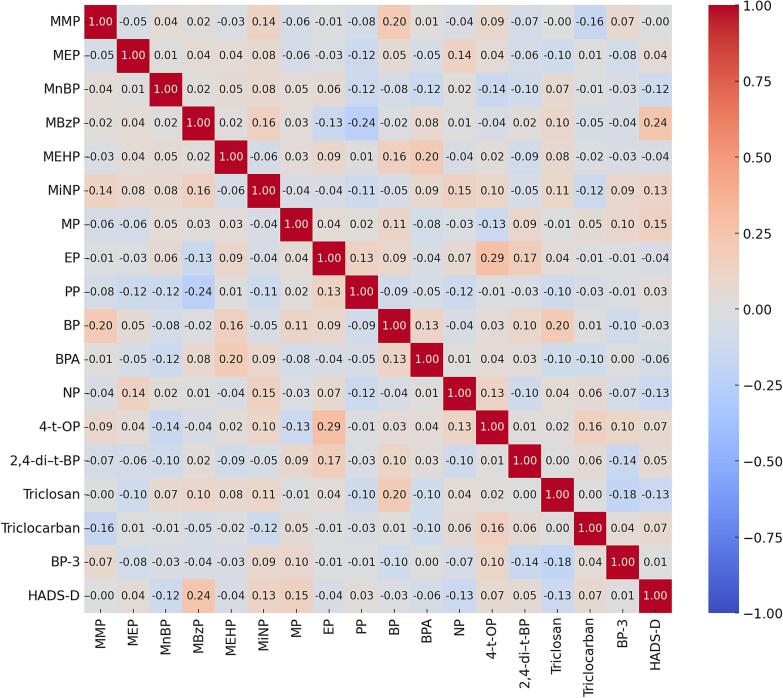
Correlation matrix showing the relationships between 17 endocrine-disrupting chemicals (EDCs) and Hospital Anxiety and Depression Scale–Depression subscale (HADS-D) scores. Each cell in the matrix represents the Pearson correlation coefficient (*r*) between two variables, with values ranging from −1 to 1. Positive correlations (in red) indicate that higher levels of an EDC are associated with higher HADS-D scores, while negative correlations (in blue) suggest that higher levels of an EDC are associated with lower HADS-D scores. The intensity of the color corresponds to the strength of the correlation. This analysis highlights specific EDCs that may be linked to depressive symptoms. Abbreviations: MMP, monomethyl phthalate; MEP, monoethyl phthalate; MnBP, mono-n-butyl phthalate; MBzP, monobenzyl phthalate; MEHP, mono-(2-ethylhexyl) phthalate; MiNP, monoisononyl phthalate; BPA, bisphenol A; NP, nonylphenol; 4-t-OP, 4-*tert*-octylphenol; 2,4-di-t-BP, 2,4-di-*tert*-butylphenol; MP, methylparaben; EP, ethylparaben; PP, propylparaben; BP, butylparaben; BP-3, benzophenone-3. (For interpretation of the references to color in this figure legend, the reader is referred to the web version of this article.)

The correlation matrix analysis revealed varying degrees of association between the 17 EDCs and HADS-D scores (Fig. 4). Notably, MBzP demonstrated a moderate positive correlation with HADS-D scores (*r* = 0.24), suggesting that higher levels of this chemical may be associated with increased depressive symptoms. In contrast, MnBP, Triclosan, NP, and BPA exhibited negative correlations with HADS-D scores (*r* = -0.12, −0.13, −0.13, and −0.06, respectively). Other chemicals, such as MiNP (*r* = 0.13) and MP (*r* = 0.15), showed weaker positive correlations, indicating a less pronounced association with depressive symptoms.

Significantly associated with higher HADS-D scores were increased MEHP levels, as shown in Model 1 (β ± SE: 0.130 ± 0.047, *p* = 0.003, adjusted for age and gender) and Model 2 (β ± SE: 0.139 ± 0.050, *p* = 0.006, adjusted for the 17 EDCs, age, gender, and diabetes) through multiple linear regression analysis (Table 3).

### 3.4 EDCs level, exposure and likelihood of depression symptoms

Binary logistic regression analyses were conducted on eleven EDCs—MnBP, MBzP, MEHP, MiNP, MP, EP, BPA, NP, BP-3, 4-t-OP, and triclosan—representing phthalates, parabens, and phenols, alongside several clinically relevant characteristics, as detailed in Table 4. MBzP demonstrated a consistently significant association with the occurrence of HADS-D scores ≥ 8 in univariate analysis (OR: 1.090, 95 % CI: 1.015–1.170, *p* = 0.018) and in multivariable analyses, both in Model 1 (OR: 1.092, 95 % CI: 1.015–1.175, p = 0.018, adjusted for age and gender) and Model 2 (OR: 1.150, 95 % CI: 1.036–1.278, *p* = 0.009, adjusted for eGFR, vitamin D, BMI, age, gender, and diabetes). Additionally, higher MP levels, diabetes, higher BMI, and younger age were found to be associated with the occurrence of HADS-D scores ≥ 8 in Model 2 (OR: 1.008, *p* = 0.001; OR: 1.007, *p* = 0.004; OR: 1.282, *p* = 0.004; OR: 0.912, *p* = 0.010).

**Table 4:** Univariate And Multivariable Analysis of the EDCs to associate HADS-D scores ≥ 8.

	Univariate			Multivariable, Model 1			Multivariable, Model 2		
	Crude OR	95 % CI	*p* value	Adjusted OR	95 % CI	*p* value	Adjusted OR	95 % CI	*p* value
MnBP	0.980	0.928–1.034	0.454	0.980	0.929–1.035	0.472	0.986	0.915–1.061	0.701
MBzP	1.090	1.015–1.170	0.018*	1.092	1.015–1.175	0.018*	1.150	1.036–1.278	0.009*
MEHP	0.979	0.879–1.091	0.703	0.967	0.867–1.079	0.550	0.969	0.841–1.118	0.668
MiNP	1.059	0.983–1.141	0.131	1.049	0.972–1.131	0.220	1.113	0.997–1.241	0.056
MP	1.005	1.001–1.008	0.004*	1.005	1.002–1.008	0.003*	1.008	1.003–1.013	< 0.001*
EP	1.006	0.989–1.024	0.477	1.005	0.988–1.023	0.538	0.999	0.974–1.026	0.964
BPA	1.000	0.997–1.003	0.782	0.917	0.745–1.129	0.414	0.881	0.651–1.192	0.411
NP	0.362	0.039–3.353	0.371	0.307	0.032–2.921	0.305	0.299	0.014–6.171	0.434
4-t-OP	3.310	0.233–47.007	0.377	3.364	0.226–50.093	0.379	29.735	0.550–1607.53	0.096
Triclosan	0.996	0.988–1.005	0.395	0.997	0.989–1.005	0.458	0.994	0.983–1.005	0.292
BP-3	1.000	0.997–1.003	0.782	1.000	0.997–1.003	0.919	0.998	0.994–1.002	0.323
Age	0.975	0.944–1.007	0.122	—	—	—	0.912	0.851–0.978	0.010*
Gender	1.245	0.500–3.098	0.638	—	—	—	0.253	0.053–1.203	0.084
Diabetes	1.000	0.301–3.321	1.000	1.199	0.347–4.144	0.774	1.007	1.081–1.521	0.004*
eGFR	1.015	0.992–1.038	0.201	1.010	0.986–1.035	0.422	0.997	0.961–1.035	0.889
Vitamin D	1.018	0.973–1.065	0.436	1.037	0.986–1.090	0.159	1.080	1.008–1.158	0.028*
BMI	1.071	0.975–1.177	0.153	1.089	0.988–1.201	0.086	1.282	1.081–1.521	0.004*

Abbreviations: EDCs, endocrine-disrupting chemicals; HADS-D, Hospital Anxiety and Depression Scale − depression subscale; OR, odds ratio; CI, confidence interval; MnBP, Mono-(n-butyl) phthalate; MBzP, Monobenzyl phthalate; MEHP, Mono-(2-ethylhexyl) phthalate; MiNP, Monoisononyl phthalate; MP, Methylparaben; EP, Ethylparaben; BPA, bisphenol A; NP, Nonylphenol; 4-t-OP, 4-*tert*-Octylphenol; BP-3, benzophenone-3; eGFR, estimated glomerular filtration rate; BMI, body-mass index.

Model 1: Asjustment for age and gender, Model 2: Adjustment for all variables.

*: *p* value < 0.05.

**Fig. 5:**
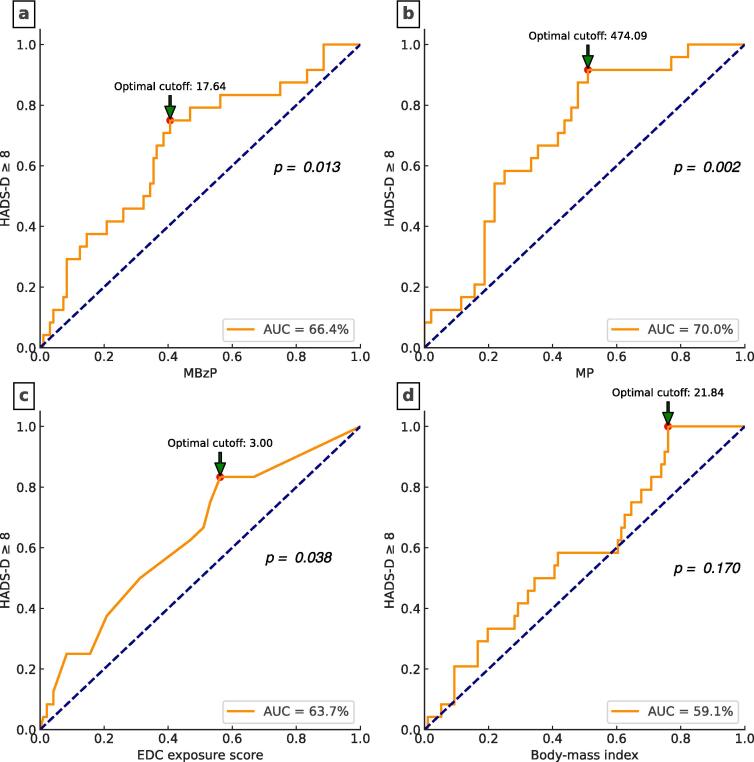
This figure shows the receiver operating characteristic curves for four predictors in relation to the occurrence of a HADS-D score of 8 or higher, indicating clinically significant depression. The predictors evaluated are (Panel A) monobenzyl phthalate (MBzP), (Panel B) methylparaben (MP), (Panel C) endocrine-disrupting chemical (EDC) exposure score, and (Panel D) body-mass index (BMI). The area under the curve (AUC) for each predictor is presented as a percentage, reflecting the ability of each predictor to discriminate between participants with and without significant depressive symptoms. The optimal cutoff points for each predictor, determined using the Youden index, are marked with a red dot on each curve and annotated in the figure. The corresponding AUCs are as follows: MBzP (66.4%, Panel A), MP (70.0%, Panel B), EDC exposure score (63.7%, Panel C), and BMI (59.1%, Panel D). (For interpretation of the references to color in this figure legend, the reader is referred to the web version of this article.)

Fig. 5 presents the ROC curves demonstrating the relationship between elevated MBzP levels (AUC: 66.4 %, *p* = 0.013), elevated MP levels (AUC: 70.0 %, *p* = 0.002), and high EDC exposure scores (AUC: 63.7 %, *p* = 0.038) with HADS-D scores ≥ 8. These results revealed that MBzP and MP levels above the cutoff values of 17.64 µg/g creatinine and 474.09 µg/g creatinine, respectively, along with EDC exposure scores above 3 points, are meaningly linked to the occurrence of HADS-D scores ≥ 8. These findings indicate a possible interplay between phthalate and paraben levels and behavioral or nutritional patterns associated with EDC exposure, potentially affecting the development of depressive disorders. Nonetheless, associated with HADS-D scores ≥ 8, though not reaching significance in the ROC evaluation, was a tendency for higher BMI.

## 4 Discussion

This prospective cohort study investigated the association between urinary EDC concentrations and the likelihood of depressive symptoms in a community-based population in northeastern Taiwan. This study has a number of limitations. First, due to budget limitations and insufficient urine sample volume, we randomly selected 60 subjects each from the low and high exposure groups for EDC measurement. Although the demographics and clinical characteristics of these 120 subjects were comparable to those of the full cohort of 887 participants (Che et al., 2021), the EDC data from this subset may not fully capture the characteristics of the broader cohort. Second, as an observational study, we cannot draw causal conclusions from the associations observed. Third, depressive symptoms were assessed using only the HADS-D questionnaire, without additional DSM-5-aligned tools, which may have limited diagnostic accuracy. Fourth, spot urine samples were used for EDC measurements, potentially introducing variability due to recent dietary or lifestyle factors, though the structured exposure questionnaire may have mitigated this bias. Fifth, interviewers were aware of participants receiving the EDC exposure questionnaire due to their roles in health education and event facilitation. Despite standardized training to reduce bias, differential data collection may still have occurred. Additionally, we did not assess other potential confounders, such as thyroid function or inflammation markers, which could influence mental health outcomes.

The results revealed that increased concentrations of MBzP were strongly linked to HADS-D scores ≥ 8, suggesting a greater risk of depressive symptoms. Furthermore, higher MBzP or MP levels and total EDC exposure scores were positively associated with depressive tendencies. The observations suggest an association between exposure to specific EDCs, particularly MBzP and MP, and the likelihood of depression, highlighting the potential psychological impact of EDC exposure observed in this cross-sectional study.

The Hospital Anxiety and Depression Scale (HADS) is a self-assessment tool intended to evaluate symptoms of anxiety and depression. It functions as a valid, reliable, and straightforward tool for detecting and measuring anxiety and depression (Hansson et al., 2009, Hartung et al., 2017, Michopoulos et al., 2008). Commonly used as a useful screening tool, HADS provides an assessment of depressive symptoms in patients without psychiatric disorders and aids in further care (Brennan et al., 2010, Lee et al., 2016). Although HADS-D is not substitutable as a definitive diagnostic tool for anxiety or depressive disorders, numerous studies have demonstrated its high internal consistency, stability, and validity for detecting depressive disorder, showcasing strong psychometric properties (Brehaut et al., 2020, Hartung et al., 2017, Lee et al., 2016, McKenzie et al., 2018). This establishes HADS-D as a dependable tool for clinicians to assess the extent of depression in patients. Additionally, HADS-D is suggested to be capable of identifying depressive disorder in individuals with physical illness (Hartung et al., 2017, Lee et al., 2016, Westhoff-Bleck et al., 2020). Higher HADS-D scores have also been found to be related to a greater chance of a subsequent diagnosis of depressive disorders (Lee et al., 2016, McKenzie et al., 2018).

Depression, characterized by neurological dysregulation, impairs the brain's ability to modulate emotions (Hasler, 2010, Tubbs et al., 2020). Consistent with previous studies, our research demonstrates an association between depression and exposure to EDCs, as reflected in higher HADS-D scores. Participants with elevated EDC exposure scores also exhibited higher HADS-D scores. Additionally, PCA of 17 EDCs and HADS-D scores revealed that specific patterns of EDC exposure were linked to higher levels of depressive symptoms (Kajta and Wojtowicz, 2013, Kim et al., 2016, Segovia-Mendoza et al., 2022, Xu et al., 2015, Zuo et al., 2014). This finding reinforces a possible connection between EDC exposure and mental health outcomes and highlights the importance of considering cumulative exposure to multiple EDCs rather than focusing on individual chemicals in isolation.

Participants with higher HADS-D scores also exhibited elevated insulin levels, and this correlation reached statistical significance. This result is consistent with evidence suggesting a link between depression and insulin resistance (Bai et al., 2018, Fernandes et al., 2022, Watson et al., 2018). The exposure to EDCs, linked to impaired insulin sensitivity and inflammation, may partly explain this relationship (Howard, 2018, Lin and Yin, 2023, Rotondo and Chiarelli, 2020). These findings highlight a potential link between psychological and metabolic health, warranting further investigation..

Among the 17 EDCs, MBzP and MP showed a near-significant trend of increasing concentrations with higher HADS-D scores. Linear and multiple regression analyses revealed a significant positive correlation between MBzP levels and HADS-D scores, with MBzP emerging as a predictor of elevated scores. MP was associated with HADS-D scores ≥ 8 in one model. ROC curve analysis further indicated that elevated levels of MBzP, MP, and overall EDC exposure were linked to a higher risk of depression. Notably, our findings demonstrate this association in a cohort of relatively healthy community residents, differing from previous studies that often focused on clinical populations or those with preexisting conditions. Thus, our study suggests that both MBzP and MP may be associated with an increased risk of developing depressive symptoms in general community settings.

Several prior studies have explored the relationship between EDCs and depression, identifying a significant association between them and emphasizing the potential negative impact of EDCs on mental health (Kim et al., 2016, Lee et al., 2018, Lin et al., 2023, Segovia-Mendoza et al., 2022). Most of these studies employed cross-sectional designs, assessing EDC exposure levels through chemical concentrations in urine samples. Some studies focused on older populations, while others included individuals with diagnosed psychiatric conditions (Kim et al., 2016, Lee et al., 2018, Segovia-Mendoza et al., 2022). Notably, the majority of these investigations were conducted in North America, encompassing diverse populations, primarily African American and Mexican American participants. In contrast, the present study involves adults aged 18 and older, predominantly of Asian descent, providing a more homogeneous study population. As a community-based study, all participants were generally healthy, and it is likely that the prevalence of depression was lower compared to those with severe physical illnesses. However, environmental EDC exposure, which is known to elicit neurobehavioral effects, may contribute to an increased risk or severity of depressive symptoms.

## 5 Conclusions

This study identified a significant link between urinary EDCs, specifically MBzP and MP, and depressive symptoms. Higher levels of MBzP and MP were associated with increased HADS-D scores, indicating a potential correlation between these EDCs and mental health. These findings underscore the importance of considering environmental factors, such as EDC exposure, in exploring the contributing factors of depression. Future research should focus on longitudinal follow-up studies and repeated EDC assessments to better elucidate causal relationships and understand how EDCs affect mental health outcomes.

## Declaration of competing interest

The authors declare the following financial interests/personal relationships which may be considered as potential competing interests: Reports a relationship with that includes: Has patent pending to. If there are other authors, they declare that they have no known competing financial interests or personal relationships that could have appeared to influence the work reported in this paper.

## Data Availability

The authors do not have permission to share data.
