# Supplementary material for: Community-based insights into the connection between endocrine-disrupting chemicals and depressive symptoms
Source: Curr Res Toxicol. 2025 Feb 25;8:100225. doi: 10.1016/j.crtox.2025.100225 (PMC11919602; doi:10.1016/j.crtox.2025.100225)
Supplement: Supplementary Data 1 [file mmc1.docx]

**Table S1**

| Endocrine-Disrupting Chemicals Exposure Screen Interview | | | | |
| --- | --- | --- | --- | --- |
|  | less than one day per week  ***0 point*** | 1-2 days per week  ***1 point*** | 3-4 days per week  ***2 points*** | 5-7 days per week  ***3 points*** |
| How often do you use plastic bags for hot food? |  |  |  |  |
| How often do you use plastic tableware? |  |  |  |  |
| How often do you consume packaged processed foods? |  |  |  |  |
| Do you use plastic wrap for microwaving, steaming, or packaging oily food at home? |  |  |  |  |
| On how many days per week do you drink a beverage in a commercially available plastic cup? |  |  |  |  |
| Do you use fragranced bathroom products? |  |  |  |  |
| Do you use plastic floor mats in your home? |  |  |  |  |
| TOTAL SCORES |  |  |  |  |
| The questionnaire was designed by Community Medicine Research Center, Chang Gung Memorial Hospital, Keelung Branch 2018 | | | | |

**Table S2**

**Hospital Anxiety and Depression Scale (HADS)**

**Tick the box beside the reply that is closest to how you have been feeling in the past week.**

**Don’t take too long over you replies: your immediate is best.**

| **D** | **A** |  | **D** | **A** |  |
| --- | --- | --- | --- | --- | --- |
|  |  | **I feel tense or 'wound up':** |  |  | **I feel as if I am slowed down:** |
|  | 3 | Most of the time | 3 |  | Nearly all the time |
|  | 2 | A lot of the time | 2 |  | Very often |
|  | 1 | From time to time, occasionally | 1 |  | Sometimes |
|  | 0 | Not at all | 0 |  | Not at all |
|  |  |  |  |  |  |
|  |  | **I still enjoy the things I used to**  **enjoy:** |  |  | **I get a sort of frightened feeling like**  **'butterflies' in the stomach:** |
| 0 |  | Definitely as much |  | 0 | Not at all |
| 1 |  | Not quite so much |  | 1 | Occasionally |
| 2 |  | Only a little |  | 2 | Quite Often |
| 3 |  | Hardly at all |  | 3 | Very Often |
|  |  |  |  |  |  |
|  |  | **I get a sort of frightened feeling as if**  **something awful is about to**  **happen:** |  |  | **I have lost interest in my appearance:** |
|  | 3 | Very definitely and quite badly | 3 |  | Definitely |
|  | 2 | Yes, but not too badly | 2 |  | I don't take as much care as I should |
|  | 1 | A little, but it doesn't worry me | 1 |  | I may not take quite as much care |
|  | 0 | Not at all | 0 |  | I take just as much care as ever |
|  |  |  |  |  |  |
|  |  | **I can laugh and see the funny side**  **of things:** |  |  | **I feel restless as I have to be on the**  **move:** |
| 0 |  | As much as I always could |  | 3 | Very much indeed |
| 1 |  | Not quite so much now |  | 2 | Quite a lot |
| 2 |  | Definitely not so much now |  | 1 | Not very much |
| 3 |  | Not at all |  | 0 | Not at all |
|  |  | **Worrying thoughts go through my**  **mind:** |  |  | **I look forward with enjoyment to**  **things:** |
|  | 3 | A great deal of the time | 0 |  | As much as I ever did |
|  | 2 | A lot of the time | 1 |  | Rather less than I used to |
|  | 1 | From time to time, but not too often | 2 |  | Definitely less than I used to |
|  | 0 | Only occasionally | 3 |  | Hardly at all |
|  |  |  |  |  |  |
|  |  | **I feel cheerful:** |  |  | **I get sudden feelings of panic:** |
| 3 |  | Not at all |  | 3 | Very often indeed |
| 2 |  | Not often |  | 2 | Quite often |
| 1 |  | Sometimes |  | 1 | Not very often |
| 0 |  | Most of the time |  | 0 | Not at all |
|  |  |  |  |  |  |
|  |  | **I can sit at ease and feel relaxed:** |  |  | **I can enjoy a good book or radio or TV**  **program:** |
|  | 0 | Definitely | 0 |  | Often |
|  | 1 | Usually | 1 |  | Sometimes |
|  | 2 | Not Often | 2 |  | Not often |
|  | 3 | Not at all | 3 |  | Very seldom |

Pl ease check you have answered all the quest ions

Scoring:

Tot al score: Depression (D) ___________ Anxiet y (A) ______________ 0-7 = Normal

8-10 = Borderline abnormal (borderline case) 11-21 = Abnormal (case)
